# Preparation and characterization of rare earth element nanoparticles for enhanced photocatalytic degradation

**DOI:** 10.1007/s11356-023-27090-2

**Published:** 2023-05-04

**Authors:** Rasha A. El-Kholy, Heba Isawi, Ehab Zaghlool, Elsayed A. Soliman, Mostafa M. H. Khalil, Moustafa M. Said, Abd-elhameed M. El-Aassar

**Affiliations:** 1 Hydrogeochemistry Department, Desert Research Center, Cairo, Egypt; 2 Chemistry Department, Faculty of Science, Ain Shams University, Cairo, Egypt

**Keywords:** Erbium, TiO_2_, Sol–gel, Photocatalyst, Organic pollutant

## Abstract

The present work focuses on the photocatalytic degradation of methylene blue (MB) on erbium ion (Er^3+^) doped TiO_2_ under visible light. Pure TiO_2_ nanoparticles and erbium (Er^3+^) doped TiO_2_ nanocomposite (Er^3+^/TiO_2_) NCs were synthesized using the sol–gel method. The synthesized (Er^3+^/TiO_2_) NCs were characterized using Fourier transform infrared spectroscopy (FTIR), high resolution scanning electron microscopy (HR-SEM), elementary dispersive X-ray (EDX), X-ray diffraction (XRD), and X-ray photoelectron spectra (XPS), specific surface area (BET), zeta potential, and particle size. Different parameters were used to study their efficiency for the photoreactor (PR) and the synthesized catalyst. These parameters include pH of the feed solution, the rate of flow, the presence of an oxidizing agent (aeration pump), different ratios of nanoparticles, the amount of catalyst, and the concentrations of pollutants. An example of an organic contaminant was the dye methylene blue (MB). The result achieved using the synthesized nanoparticles (I) under ultraviolet light pure TiO_2_ was found to have degraded by 85%. For (Er^3+^/TiO_2_) NCs under visible light, dye removal increased with pH to a maximum of 77% degradation at pH 5. Furthermore, photocatalytic efficiency improves to 80% at 40 rpm (3 l/h) low motor speed. The degradation efficiency decreased to 70% when the MB concentration was increased from 5 to 30 mg/L. When oxygen content was increased using an air pump, and deterioration reached 85% under visible light, it improved performance.

## Introduction

In recent years, contamination of the environment, such as water and air pollution, has become a worldwide crisis (Reszczyńska et al. 2015). Due to the rapid increase in global population and increased release of harmful chemicals into the atmosphere due to the expansion of industry in emerging countries, natural air and water quality issues have become more complicated. As a result, researchers must focus on developing cost-effective, non-toxic, and mostly stable materials that may be used in advanced remediation technologies (Hamal 2009). The use of photocatalytic oxidation in water treatment, air purification, and sunlight consumption has drawn various attentions (Shi et al. 2012). Photocatalytic oxidation, which was developed in the 1970s, has attracted great deal of interest, mainly when utilized under sunlight (Teh and Mohamed 2011). TiO_2_ as a photocatalyst has been the subject of numerous investigations over the past two decades due to its attractive properties and potential in the treatment of environmental pollutants (Lin and Jimmy 1998). Using solar radiation or ultraviolet (UV) light as an energy source, TiO_2_ promotes the breakdown and removal of several contaminants. In addition to its strong photocatalytic activity, TiO_2_ is chemically inert, non-toxic, photo-corrosion resistant, and an inexpensive nanomaterial (Shah and Rather 2021). As a photocatalyst for environmental remediation, titanium dioxide (TiO_2_) is commonly used because of its remarkable stability and oxidative power. Although the two distinct crystal forms of TiO_2_ (rutile and anatase) are extensively used in photocatalytic applications, the anatase phase exhibits greater photocatalytic activity (Linsebigler et al. 1995; Syarif et al. 2002; Khakpash et al. 2012). As a result, traditional TiO_2_ photocatalysts cannot absorb visible light due to their high band gap approximately 3.2 eV for anatase. TiO_2_ must be made visible light-sensitive, as the solar energy contains 48% of visible light (Zhang et al. 2011). Researchers are now concentrating their efforts on the creation of new materials that can be used in place of traditional TiO_2_ as a green energy source (Obregón et al. 2013; Kubacka et al. 2012). When TiO_2_ is combined with other materials, its thermo-mechanical and optoelectronic properties may be improved (Murray et al. 1993; Zhang et al. 2000; Patil et al. 2017). Therefore, numerous studies have focused on enhancing the photoactivity of TiO_2_ through the introduction of mid-band gap donors or acceptors (Gu et al. 2007; Hsieh et al. 2009). Doping TiO_2_ with anions (Silveyra et al. 2005; Sano et al. 2008), transition metals (Dvoranová et al. 2002), and rare earth metals (Yang et al. 2002) are promising methods to shift the absorption edge and enhance photocatalytic activity (Khakpash et al. 2012).

In recent years, TiO_2_ doped with rare earth (RE) metals has proved to be an efficient method to improve the photocatalytic properties of TiO_2_ because the f-orbitals of the lanthanide ions can form complexes with various Lewis bases and thus, concentrate the substrates onto the TiO_2_ surface (Bellardita et al. 2011; Reszczyńska et al. 2015). In addition, doping with rare-earth ions can reduce the crystallite size and increase the surface area of TiO_2_, both of which improve the adsorption ability of TiO_2_ for organic contaminants (Liang et al. 2006; Venkatachalam et al. 2019; Talane et al. 2018). Most recently, interest in luminous rare-earth ions focused on Er^3+^ because of its distinct electrical and optical features (Reszczyńska et al. 2014). In the present study, the sol–gel method was utilized to dope erbium ions into TiO_2_ structures with variable amounts, resulting in pure TiO_2_ and a range of erbium-doped TiO_2_ nanocomposites (Er^3+^/TiO_2_) NCs catalysts. The ability to generate high-purity nano-sized crystalline powders at low temperatures additionally its low cost and reproducibility make the sol–gel process an excellent choice for industrial applications. The erbium ion was chosen due to its potential absorption of light in the visible spectrum. Additionally, the erbium ion possesses unique luminous characteristics. This paper will focus on the exceedingly stable and active TiO_2_-based composites. These catalysts were studied for their structural, morphological, molecular functional and optical absorption properties and their effect on the photocatalytic degradation of MB. In aqueous suspension, the effect of the synthesized pure TiO_2_ and (Er^3+^/TiO_2_) NCs catalysts on the adsorption, degradation, and mineralization of MB were examined.

The doping of rare-earth ions can reduce the recombination chance of photogenerated electron–hole pairs, which leads to the improvement of photocatalytic efficiency (Regulska et al. 2022). The MgO coupled LaFeO_3_: Er^3+^ composite as a photocatalyst has higher CO_2_ adsorption capacity and is more effective in the separation of photo-generated electron–hole pairs compared to the pure LaFeO_3_, which are contributed to the improvement of CO_2_ photocatalytic performance (Li et al. 2020).

The synthesized pristine TiO_2_ and (Er^3+^/TiO_2_) NCs were characterized using FTIR, HRSEM, X-ray-EDX, XRD, XPS, BET, zeta potential, and the particle size. Also, the efficiency of the synthetized NCs and the photoreactor (PR) were evaluated by studying the effect of different parameters. These parameters included feed water pH, flow rate, oxygen content, dosing concentrations of catalyst, doping ratios of catalyst, light intensity under visible light, and pollutant concentrations. Methylene blue was used as an example of organic pollutants. This technique develops the photocatalytic activity and solves the problem of high concentration of organic contaminants in the surface and/or groundwater.

This research aims to fabricate and design a continuous flow photoreactor for heterogeneous photocatalytic treatment of organic wastewater pollutants. The greatest advantage of this technique is lower maintenance, costs, and construction as well as it presented favorable results in the degradation of organic contaminations from wastewaters. Preparation of TiO_2_ doped with erbium via sol–gel method process aims to enhance the photocatalytic activity in sunlight.

## Experimental section

### Materials

Methylene blue was acquired from Bioworld Chemicals, USA, and utilized as received without additional purification. Chemicals including titanium (IV) isopropoxide Ti[OCH(CH_3_)_2_]_4_, Er(III) chloride hexahydrate (ErCl_3_.6H_2_O), glacial acetic acid (99%), and absolute ethanol (99.9%) were purchased from Alpha Chemika, Aldrich, Adwic and VWR(BDH) chemicals, respectively and used as received. Deionized water (DI) was used as a solvent for the thiazine dye (MB).

### Synthesis of pristine TiO_2_ and erbium-doped TiO_2_ nanocomposites (Er^3+^/TiO_2_) NCs through sol–gel method

**Fig. 1:**
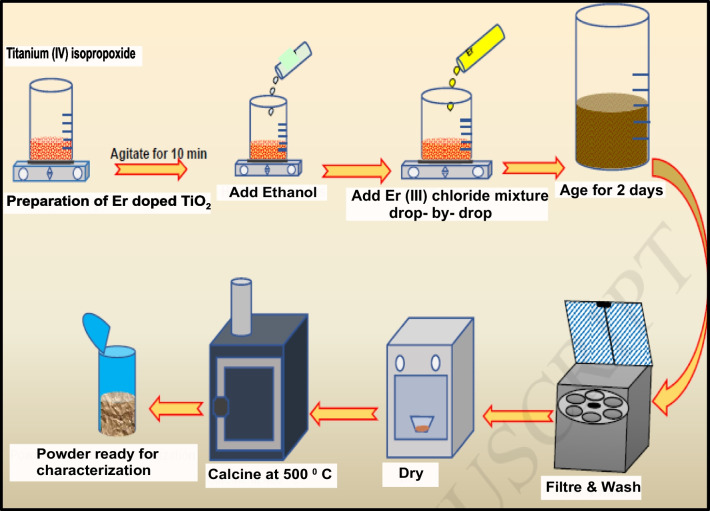
Sol–gel of erbium doped TiO_2_ nanocomposites

To make a series of (Er^3+^/TiO_2_) NCs catalysts using the sol–gel method, the following procedures were followed (Fig. 1): first, 14 ml of titanium (IV) isopropoxide [Ti(OCH)(CH_3_)_2_)_4_] was dissolved in 80 ml of pure ethanol (99.9%). The titanium (IV) isopropoxide solution was added drop-by-drop to 100 ml of a mixture containing 84 ml of 99.9% pure ethanol, 1 ml of 0.1 mol ErCl_3_.6H_2_O, and 15 ml of 99.9% pure glacial acetic acid under vigorous stirring for 2 h before the clear colloidal suspension was aged for 2 days to induce gel formation. The gel was dried and ground into powder using a vacuum at 373° K. At 773° K, the powder was calcined for 2 h. Finally, a nominal atomic doping level of 0.5% (Er^3+^/TiO_2_) NCs powder catalyst was produced. Other (Er^3+^/TiO_2_) NCs samples were also generated as 1.0% Er^3+^/TiO_2_ NCs, and 2.0% (Er^3+^/TiO_2_) NCs, using the same process. It was possible to produce pristine TiO_2_ without the use of (ErCl_3_.6H_2_O) in the process.

### Photocatalytic experiments

**Fig. 2:**
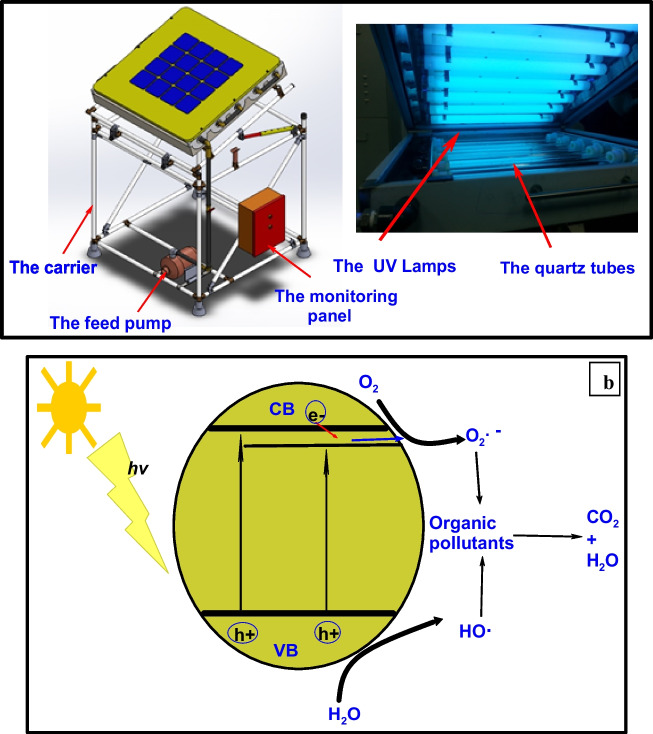
**a** Sketch diagram of the single unit of the continuous flow photoreactor. **b** The proposed photocatalytic mechanism

Photocatalytic activity of TiO_2_ and (Er^3+^/TiO_2_) NCs conducted to evaluate photo-assisted degradation of MB under ultraviolet and visible light illumination. The continuous flow photoreactor (PR) was used for the reactions (Abdel-Hameed et al. 2019). Figure 2a shows the continuous flow photoreactor (PR). All of the previously mentioned components are transported on a trolley, which includes a pollutant injection tank, a main electrical panel, a variable speed-driven feed pump, a monitoring panel (which includes flow meters, temperature gauges, and pressure gauges), and ABS plastic-coated steel pipes, a combination of connected bolts, 6 connected quartz tubes, and 7 UV lamps. A frequency generator provides the assemblage with its source of energy. Six quartz tubes are linked together to form the continuous flow photoreactor. The inner diameter of each tube is equal to 18 mm, and the outer diameter is equivalent to 20 mm. Each tube measures 52 cm in length. Even the most corrosive chemical mixes would not harm the quartz tubes linked to 50-mm-thick stainless-steel plates. The plates are covered up in a straight line along the length of the photoreactor. In the continuous flow photoreactor, the nanoparticles are fixed to the quartz tubes. Using plastic-coated steel tubing the carrier trolley was constructed and loaded with the skeleton parts. The feed flows directly over the surface at rates between 10 and 80 m^3^/h. Seven UV lamps with a specific a Sylvania UVA lamp (F40W/2FT/T12/BL368) and a 365 nm wavelength are used in the continuous flow photoreactor. When steady-state values are reached, the methylene blue photocatalytic degradation efficiency is measured for each test (after 30 min). Every 15 min, the output samples are taken. Figure 2a shows the model unit’s continuous flow photoreactor structure in detail. At a concentration of 10 parts per million (ppm), the dye was added to a known quantity of photocatalyst. An equilibrium absorption–desorption condition was achieved by vigorously stirring this solution, which had to be held in darkness before measurement (Singh et al. 2018). To be sure that the dye was destroyed by the photocatalyst, a blank experiment was carried out either without a photocatalyst or in complete darkness. After centrifuging 3 mL of the suspension, the spectrometer (Perkin-Elmer Lambda 750) measured its UV–Vis spectrum. The characteristic band absorption used to measure dye degradation is 650 nm, and the photodegradation % was calculated using this value;

$$D\left(\mathrm{\%}\right)= \frac{{C}_{0}-{C}_{\mathrm{t}}}{{C}_{0}}\mathrm{ x }100$$

where *C*_0_ is the dye’s absorption before illuminating the visible spectrum is applied, and *C*_t_ represents the dye’s absorption at interval time while being illuminated by visible light (sunlight), the intensity of sunlight was measured by a solar power meter (model: SPM-11165SD) (Devipriya and Yesodharan 2005; Liang et al. 2006).

### The estimated photocatalytic mechanism of the continuous flow photoreactor

Figure 2b shows an approximated reaction process for the photodegradation of aqueous MB catalyzed by (Er^3+^/TiO_2_) NCs by photocatalytic process. When the photocatalyst is exposed to light with a super band gap, TiO_2_ photocatalysis creates electron–hole pairs. It is anticipated that the electron will be absorbed by O_2_ and the hole will be transferred by the adsorbed hydroxide to form ^•^OH radicals on the surface. The low performance of TiO_2_ photocatalytic systems is mostly due to the high chance of electron–hole recombination. TiO_2_ crystal lattice faults alter the band gap energy, but rare-earth ions create a new electronic state in the middle of the band gap (Xie and Yuan 2003; Xu et al. 2002; Silva et al. 2009). This presumed mechanism is based in part on previously published research on the genesis of ^•^OH radicals formed during the photocatalytic reaction (Shi et al. 2012; Elkholy et al. 2020).

There is a possibility that a dual mechanism, as proposed by Obregón et al. (2013). In the presence of visible light, the production of electron–hole pairs is enhanced when Er^3+^ is doped into mesoporous TiO_2_. When Er^3+^-doped TiO_2_ is exposed to light, the excited electrons change the Er^3+^ to Er^2+^. This makes it easier for O_2_ to stick to the TiO_2_. This makes TiO_2_ better at separating electrons and holes and makes it more effective as a photocatalyst. Er^3+^ cations and flaws in the anatase structure of TiO_2_ are to blame for the rise in photocatalytic activity (Huang et al. 2013). As a result of the presence of Er^3+^ in the system, it has been suggested (Lee et al. 2013) that the presence of Er^3+^ can lead to the formation of oxygen vacancies below the conduction band that serve as a trap for electrons passing via electronic transition gap (Fig. 1 b).

### Nanomaterials characterizations and performance assessment

The synthesized nanoparticles were characterized by ATR-FTIR Spectroscopy, THERMO NICLOT, 50. The phase identification of the prepared nanoparticles was carried out by X-ray diffraction (XRD) at room temperature using a D8 advance diffractometer with a Cu target (*λ* = 1.54 Å) with the accelerating voltage of 40 kV and the emission current of 40 mA, BRUKER CO, Germany. The surface and structural morphology of the prepared nanoparticles were characterized by high-resolution scanning electron microscopy (HR-SEM) and elementary dispersive X-ray (EDX) analysis (FEI Quanta FEG 250 instrument with EDX Analyzer facility at 25 °C) to conduct elemental investigation. A monochromatic X-ray Al K-alpha radiation (K-ALPHA, Thermo Fisher Scientific, USA) with a spot size of 400 mm at a pressure of 10–9 mbar with a complete spectrum pass energy of 200 eV and a narrow spectrum of 50 eV was used to record the XPS spectra. Ultraviolet–visible spectrum analysis revealed the formation of nanoparticles. Shimadzu UV–2600 UV–VIS double beam spectrophotometer was used to record the absorbance spectra at wavelengths ranging from 200–800 nm. Dynamic light scattering (DLS) was used to measure the Zeta potential and particle size. It was collected on the Nicomp Nano Z3000 system and is available for size and zeta potential analysis. The specific surface area (BET technique), the specific pore volume (BJH method), and the average pore diameter (BJH method) of the catalysts were all measured by the Quantachrome NOVA-2000 sorption analyzer (USA) at 77 K using nitrogen adsorption–desorption. The UV/visible spectrophotometer, model 300, Unicam, England, was used to measure the absorption of the methylene blue solution to get the proper wavelength. The analytical samples were obtained from the suspension solution at the specified time intervals and centrifuged at 4000 rpm for 10 min before being analyzed with a UV/visible spectrophotometer.

## Result and discussion

### Characterizations of the nanomaterials

**Fig. 3:**
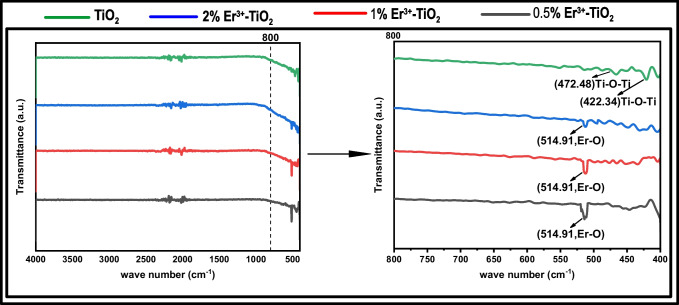
The FT-IR spectra of pure TiO_2_ and Er.^3+^/TiO_2_NCs with different ratio. All the experiments were done at room temperature (25 °C)

The FT-IR transmittance spectra of pristine TiO_2_ and (Er^3+^/TiO_2_) NCs with varying Er^3+^ concentrations are shown in Fig. 3. All FT-IR transmittance spectra, as seen in this figure, consist of three different bands. A band between 400 and 800 cm^−1^ is found in the low wavenumber range. At 472 cm^−1^ and 422 cm^−1^, the absorption peaks are owing to the stretching vibrations of Ti–O–Ti. Additionally, the Er–O bond’s stretching vibration is focused at 514 cm^−1^, which coincides with the initial peak of the Ti–O–Ti vibration (Venkatachalam et al. 2019). The strength of the peaks decreases as the erbium dopant concentration increases (Talane et al. 2018).

**Fig. 4:**
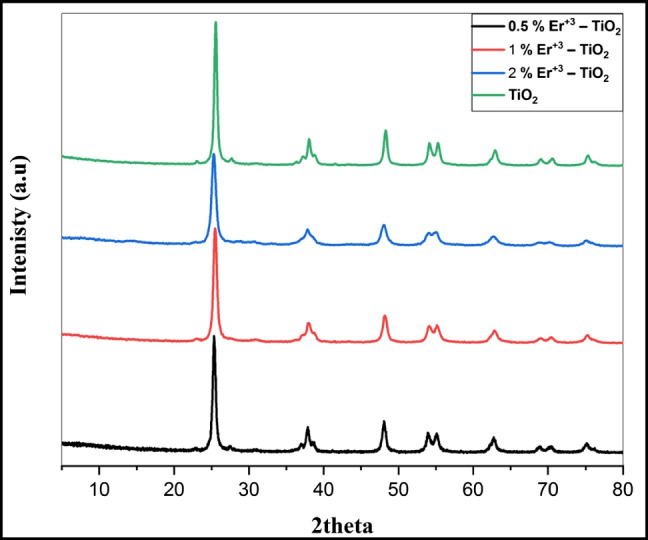
X-ray diffraction patterns of pure TiO_2_ and Er^3+^/TiO_2_NCs with different ratios

X-ray diffraction (XRD) was used to analyze the crystal structure of pristine TiO_2_ and (Er^3+^/TiO_2_) NCs catalyst powders with different Er^3+^ ion concentrations, as shown in Fig. 4. The nanoparticles’ XRD configurations were found to be almost identical. As illustrated in Fig. 4, the intense peaks at 25°, 38°, 48°, 53°, 55°, and 62°, respectively, define TiO_2_ tetragonal anatase structure (Manoharan et al. 2014). As the Er^3+^ concentration increased, diffraction peak intensity reduced. The Scherrer formula was used to estimate the diameters of (Er^3+^/TiO_2_) NCs crystallites from XRD data:

$$D=K\lambda /\upbeta \mathrm{cos}\uptheta$$

*K* = 0.9, *λ* is the X-ray wavelength (0.1540 nm), *β* is the diffraction peak’s corrected half-width, and *θ* is the Bragg’s angle (°) (Manoharan et al. 2014). The results showed that the crystallite size was reduced by increasing the Er^3+^ dosage. It suggested that erbium ion doping might prevent the transition from anatase to rutile, increase thermal stability, and limit the formation of crystallites. The Scherrer formula was used to calculate the crystallite sizes of all samples based on the FWHM of the most intense anatase peak in Table 1. The crystallite size was determined to be 28.99 nm for pristine TiO_2_ but decreased to 25.37, 23.60, and 21.02 nm for 0.5, 1, and 2% (Er^3+^/TiO_2_) NCs, respectively, indicating that the outline of Er^3+^ ions into the TiO_2_ lattice may impede crystallite growth owing to dopant cation isolation at the grain boundary (Pan et al. 2011; Jeon and Braun 2003).

**Table 1:** The full width at half maximum (FWHM) of the most intense anatase peak and the calculated crystallite size of pristine TiO_2_ and (Er^3+^/TiO_2_) NCs

Er^3+^ doping content (mol/mol)				
	0.0%	0.5%	1.0%	2%
Crystal structure	Anatase	Anatase	Anatase	Anatase
Crystallite size (nm)	28.99	25.37	23.60	21.02
FWHM	0.55784	0.6548	0.69885	0.76523
Angle 2 θ	25.40	25.37	25.40	25.20
*d* value (Angstrom)	3.50323	3.50783	3.50323	3.53102
Intensity (count)	8.48	7.4	5.78	5.49

**Fig. 5:**
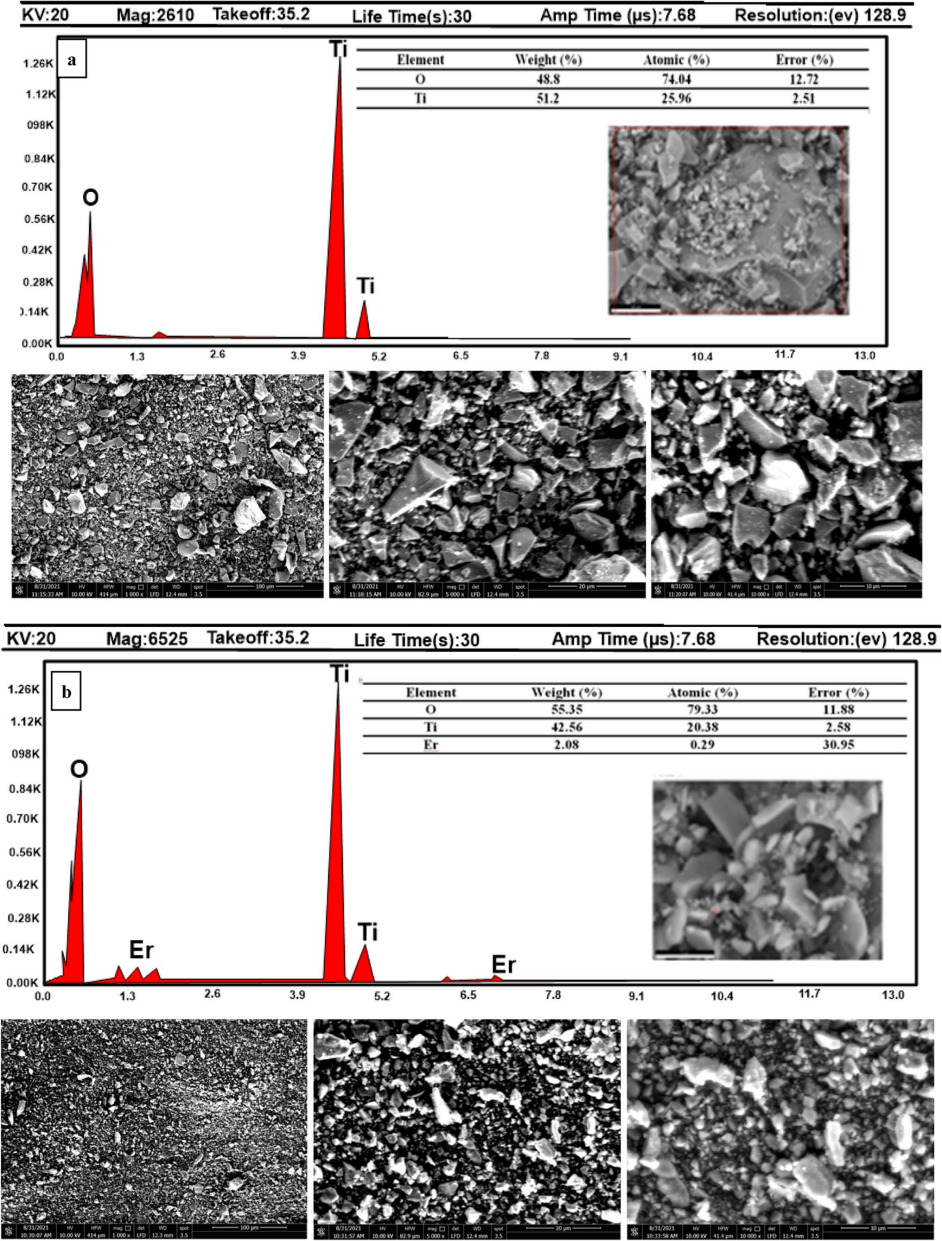

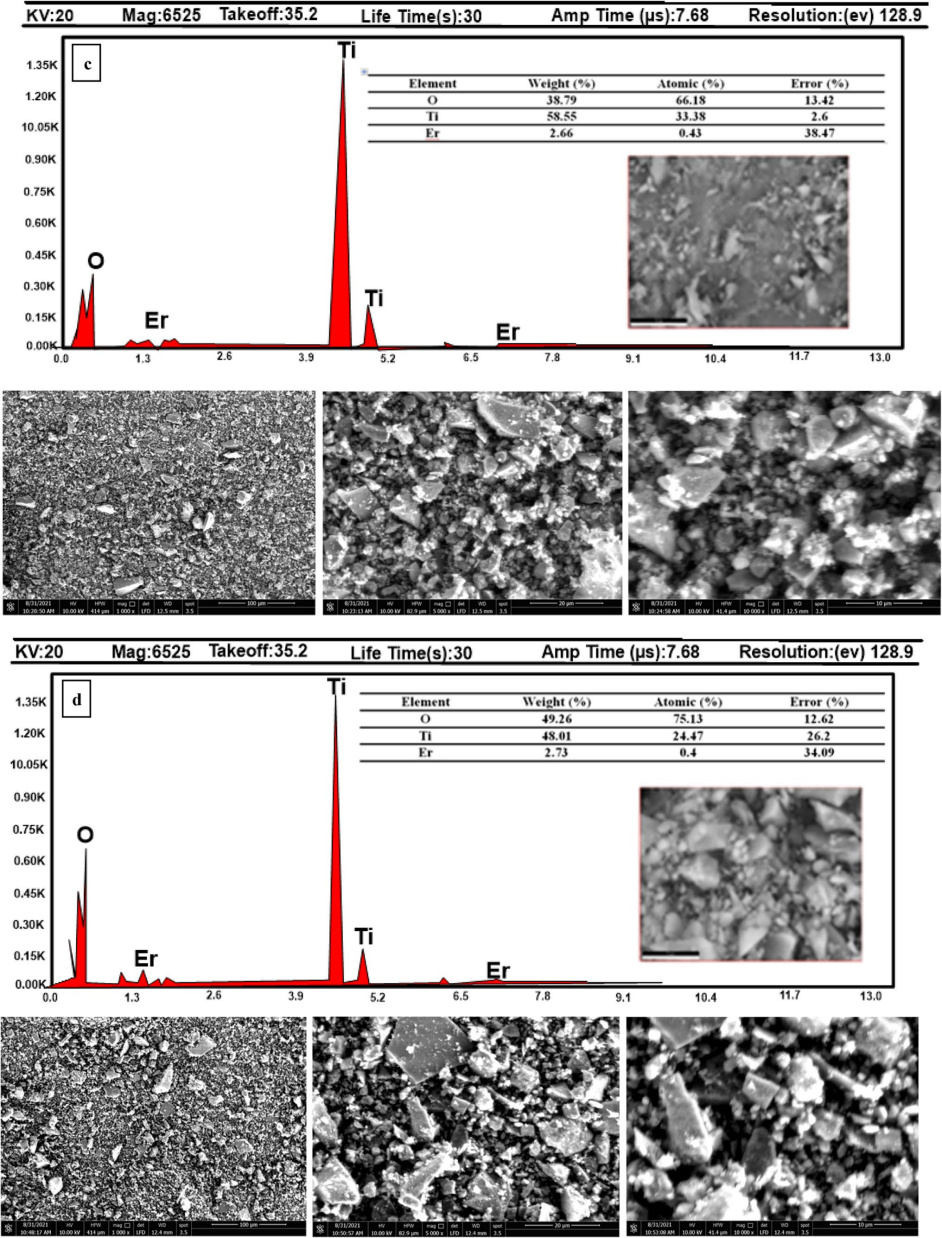
SEM image and EdX analysis of **a** pure TiO_2_, **b** 0.5% (Er^3+^/TiO_2_) NCs, **c** 1% (Er^3+^/TiO_2_) NCs, and **d** 2% (Er^3+^/TiO_2_) NCs

The surface and structural morphology of the prepared pristine TiO_2_ and (Er^3+^/TiO_2_) NCs samples were characterized by using high resolution scanning electron microscopy (HR-SEM) and energy dispersive X-ray (EDX) analysis. Figures 5 a–d illustrate the SEM images of the synthesized pristine TiO_2_ and (Er^3+^/TiO_2_) NCs with different Er^3+^ concentrations. Images were taken at different magnifications (from 1000 to 10,000). When investigating pristine TiO_2_, it was revealed that the particles crystallized into irregular forms and agglomerated. Even after varied quantities of Er^3+^ ions were added to TiO_2_, the particle-like features remained. EDX elemental maps were used to establish the occurrence and dispersal of Ti, Er, and O elements. According to the EDX results shown in Fig. 5, titanium (Ti) and oxygen (O) atoms are present in the undoped sample, whereas erbium (Er) atoms are present in the doped samples, demonstrating that Er^3+^ ions have been incorporated into TiO_2_. The atomic fraction of Er^3+^ ions in the doped nanomaterials is estimated to be 0.29 at 0.5% (Er^3+^/TiO_2_) NCs and 0.43 at 1% (Er^3+^/TiO_2_) NCs and 0.4 at 2% (Er^3+^/TiO_2_) NCs (Talane et al. 2018; Mondal et al. 2018). The presence of titanium was determined by EDX analysis of pristine TiO_2_. The EDX profile revealed a significant TiO_2_ signal in addition to other faint signals (Srinivasan et al. 2019). In the present study, TiO_2_ nanoparticles revealed a strong absorption spectrum at 1.26 keV (usually given for *x*-axis values not *y*-axis) (Fig. 5a).

**Fig. 6:**
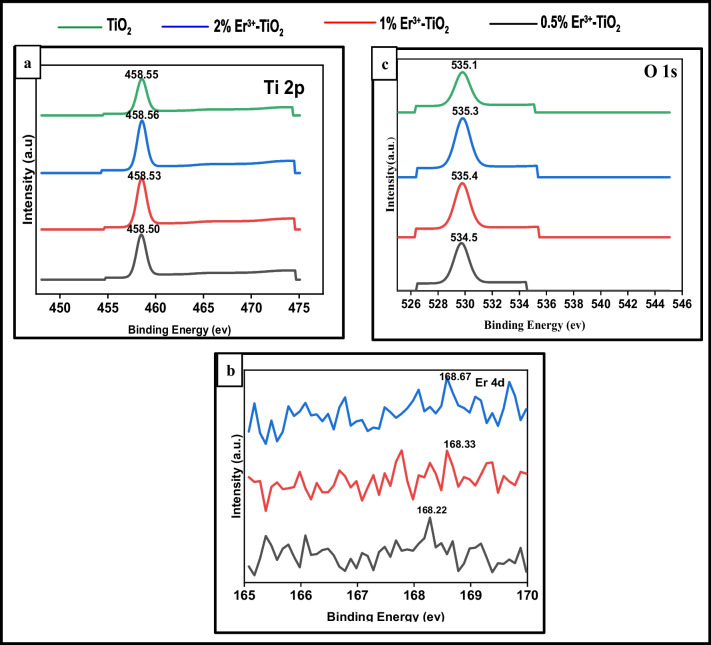
XPS spectra **a** Ti 2p, **b** Er 4d, and **c** O 1 s states of synthesized nanocomposite

The surface electronic states of Ti, Er, and O elements in pristine TiO_2_ and (Er^3+^/TiO_2_) NCs were determined by XPS analysis. Figure 6a shows the Ti 2p peaks for pristine TiO_2_, 0.5% (Er^3+^/TiO_2_) NCs, 1% (Er^3+^/TiO_2_) NCs, and 2% (Er^3+^/TiO_2_) NCs are observed at 458.55, 458.56, 458.53, and 458.50 eV (Singh et al. 2018). It is seen that the binding energy for Ti 2p rises by 0.1 eV as the binding energy rises in 0.5% (Er^3+^/TiO_2_) NCs), 1% (Er^3+^/TiO_2_) NCs, which are doped with erbium, but not in 2% (Er^3+^/TiO_2_) NCs. This shift towards Ti^3+^ is caused by the presence of Er-Ti–O. The binding energy of Ti 2p is shown to decrease when it approaches the binding energy of S2. This difference indicates the existence of Ti^3+^. Furthermore, Ti^3+^ sites decreased TiO_2_ band gap. The calcination results in the reduction of Ti^4+^ to Ti^3+^ (Santhi et al. 2020). Figure 6b shows Er 4d state of the samples. Peaks 168.22, 168.33, and 168.67 eV belong to 0.5% (Er^3+^/TiO_2_) NCs, 1% (Er^3+^/TiO_2_) NCs, and 2% (Er^3+^/TiO_2_) NCs, respectively. The shift in the peaks of 0.1 eV towards lower binding energy was observed. This could be due to interaction with Ti atoms; Er 4d includes the approximate positions of Er_2_O_3_. Figure 6c shows the O 1 s states of samples. Peaks at 535.1, 535.3, 535.4, and 535.5 eV are detected for pristine TiO_2_, 0.5% (Er^3+^/TiO_2_) NCs, 1% (Er^3+^/TiO_2_NCs), and 2% (Er^3+^/TiO_2_) NCs, respectively, which are formed by lattice oxygen in Ti–O–Ti bonds (Diebold and Madey 1996). As the concentration increased from 0.5 to 2%, there was a shift in the peaks toward greater binding energies. These shifts could be due to Erbium interaction with oxygen molecules (Sedik et al. 2017).

**Fig. 7:**
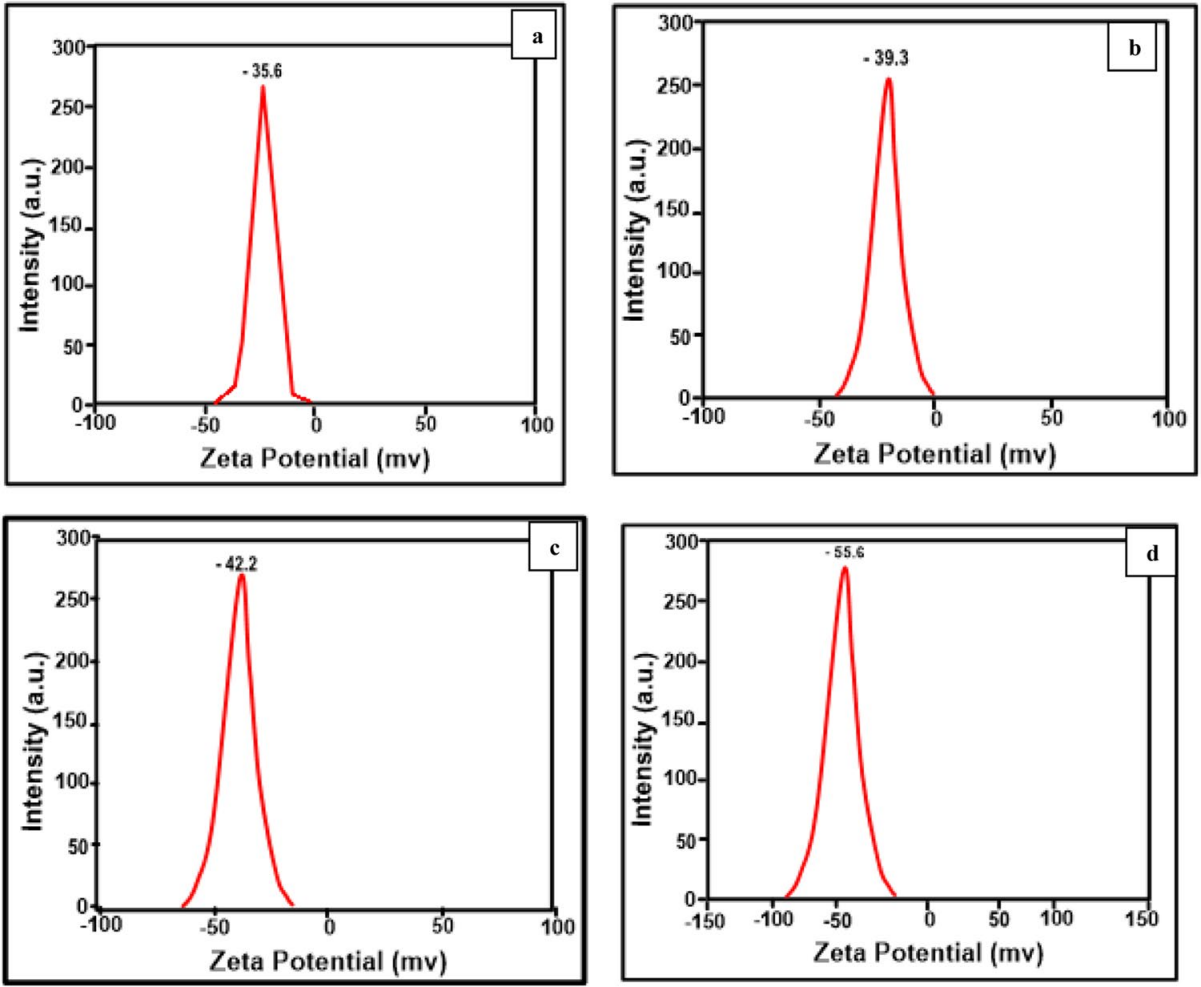
Zeta potential determination of **a** pure TiO_2_, **b** 0.5% (Er^3+^/TiO_2_) NCs, **c** 1% (Er^3+^/TiO_2_) NCs, and **d** 2% (Er^3+^/TiO_2_) NCs

The zeta potential is an important factor in mediating particle–particle interaction. When the zeta potentials are high, they will repel each other regardless of the charge sign. There is widespread agreement, however, that the term “high zeta potential” can refer to a high value in both the positive and negative senses, such as 0 to 5 rapid coagulation or flocculation, 10–30 incipient instability, 30–40 moderate stability, 40–60 good stability, and more than 60 excellent stability (Viswarupachary et al. 2017). The solution or dispersion will resist aggregation for molecules and particles that are tiny and have a low relative density to remain suspended. The Litesizer™ 500 Particle Analyzer (Anton Paar) was used to measure hydrodynamic particle size and zeta potential using the DLS (dynamic light scattering) method. To generate a homogeneous dispersion, dried powdered nanoparticles were disseminated in deionized water and sonicated for four hours in a bath sonicator. The particle size and zeta potential were measured at 25 °C with a laser wavelength of 660 nm (Shah and Rather 2021). Table 2 shows that all samples have a negative zeta potentials range from –35 to –55 mV. It is clear from these results that the dispersion medium has good stability and great dispersion of nanoparticles. This also demonstrates that the nanoparticles resisted one another and did not flocculate (Grover et al. 2013; Pandi and Gopinathan 2017; Mikolajczyk et al. 2015). The zeta potential was represented in Fig. 7.

**Table 2:** The values of zeta potential and particle size of pristine TiO_2_ and (Er^3+^/TiO_2_) NCs

Composition	Zeta potential (− mV)	Particle size (nm) mean diameter
0.5% Er^3+^–TiO_2_	39.3	36
1% Er^3+^–TiO_2_	42.2	27.8
2% Er^3+^–TiO_2_	55.6	27
TiO_2_	35.6	55.4

**Fig. 8:**
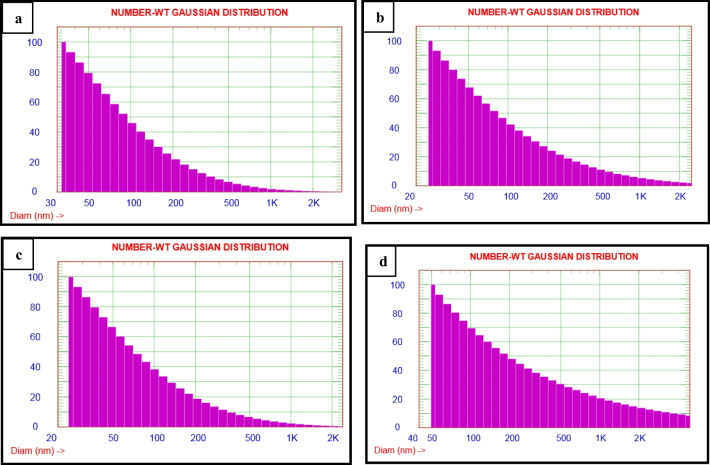
Particle size distribution of **a** pure TiO_2_, **b** 0.5% (Er^3+^/TiO_2_) NCs, **c** 1% (Er^3+^/TiO_2_) NCs, and **d** 2% (Er^3+^/TiO_2_) NCs

**Fig. 9:**
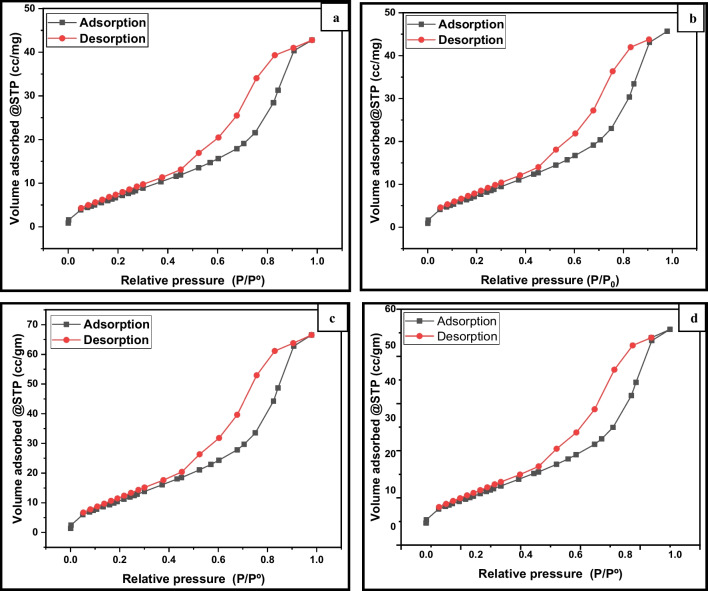
Nitrogen adsorption–desorption isothermal of **a** pure TiO_2_, **b** 0.5% (Er^3+^/TiO_2_) NCs, **c** 1% (Er^3+^/TiO_2_) NCs, and **d** 2% (Er^3+^/TiO_2_) NCs

**Fig. 10:**
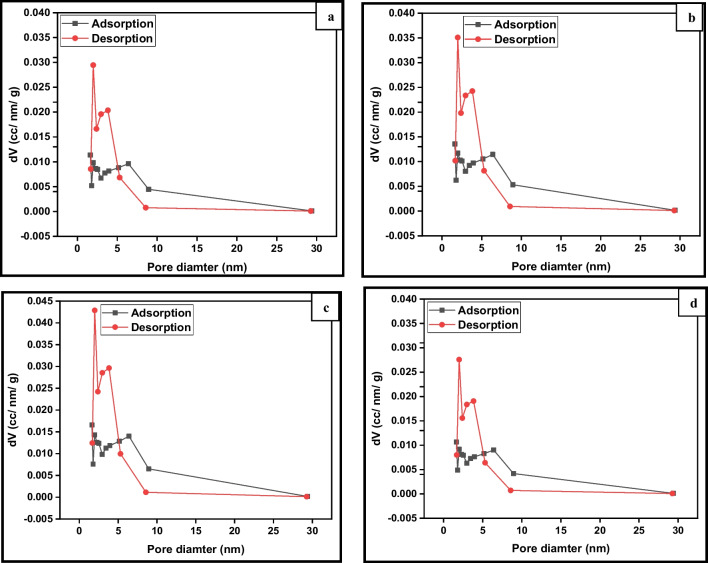
Pore size distribution plots of **a** pure TiO_2_, **b** 0.5% (Er^3+^/TiO_2_) NCs, **c** 1% (Er^3+^/TiO_2_) NCs, and **d** 2% (Er^3+^/TiO_2_) NCs

The dynamic sizes of pristine TiO_2_ and (Er^3+^/TiO_2_) NCs were measured using the DLS technique and determined to be 32.6 nm to 164 nm in size, as shown in Fig. 8. When the concentration of rare-earth ion Er^3+^ was increased, the hydrodynamic particle size of the nanocomposite was affected (Grover et al. 2013; Pandi and Gopinathan 2017; Mikolajczyk et al. 2015). Table 2 lists the values of zeta potential and particle diameter. N_2_ adsorption–desorption isotherm and Barrett–Joyner–Halenda (BJH) pore size distribution are shown in Figs. 9 and 10 for the typical samples. The isothermal curves for nitrogen adsorption and desorption are clearly defined. The isotherms reveal an increase in adsorption at low relative pressures (p/p_0_ < 0.4), which implies that the powders are mesoporous (Shi et al. 2012). At a middle relative pressure (p/p_0_ = 0.4–0.9), H1 hysteresis loops can be seen in the curves, indicating that the powders are mesoporous photocatalysts (Fig. 9).

The typical samples exhibit strong mesoporosity and pore size uniformity, with an average pore size of roughly 4.8 nm and 3.5 nm, respectively, as shown in Fig. 10, which is an isotherm type IV curve with an H3 hysteresis loop. For the erbium-doped TiO_2_ nanoparticles, the BET surface area and the pore volume are shown in Table 3. The smallest BET surface area and pore volume of 27 m^2^/g and 0.066 cm^3^/g, respectively, are found in the pristine TiO_2_ photocatalyst. The surface area and pore volume of the TiO_2_ were modified with 1 mol%, respectively, are 43 m^2^/g and 0.103 cm^3^/g. Photocatalyst adsorption is heavily dependent on organic substrate adsorption, and higher specific surface areas can improve both interfacial charge transfer and photocatalytic activity (Pan et al. 2011). Crystal formation and annealing of catalyst particles reduced the surface area (Tsumura et al. 2002).

**Table 3:** BET surface area and pore volume of Er^3+^–TiO_2_ photocatalysts

Composition	S_BET_ [m^2^/g]	Pore volume [cm^3^/g]
0.5% Er^3+^–TiO_2_	29	0.071
1% Er^3+^–TiO_2_	43	0.103
2% Er^3+^–TiO_2_	35	0.084
TiO_2_	27	0.066

**Fig. 11:**
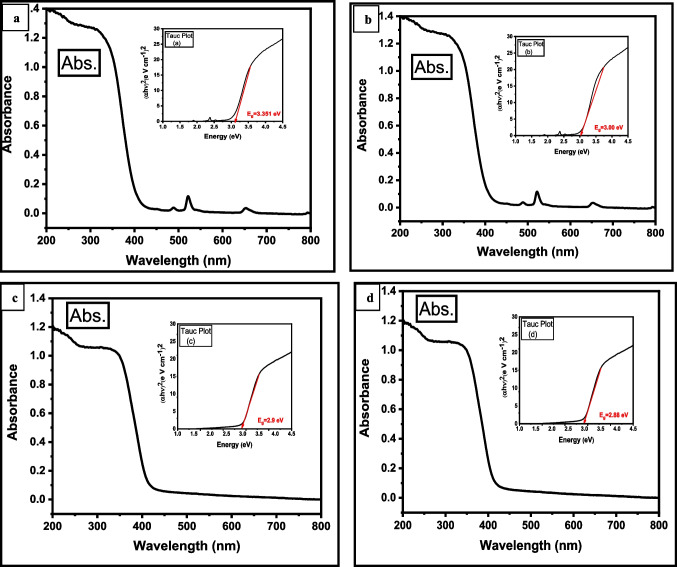
Diffuse reflectance spectra of **a** pure TiO_2_, **b** 0.5% (Er^3+^/TiO_2_) NCs, **c** 1% (Er^3+^/TiO_2_) NCs, and **d** 2% (Er^3+^/TiO_2_) NCs

A Uv–visible spectral study revealed the formation of TiO_2_ and (Er^3+^/TiO_2_) NCs in both pure and mixed forms. Shimadzu UV–2600 UV–VIS double beam spectrophotometer was used to record the absorbance spectra at wavelength 200–800 nm. When light interacts with materials, some of the light can be absorbed, transmitted, or reflected. The Uv–vis-diffuse reflectance spectrum (UV–VIS-DRS) is used to evaluate the optical properties of pristine TiO_2_ and (Er^3+^/TiO_2_) NCs, as shown in Fig. 11. The Tauc equation is used to calculate the optical band gap energy E_g_, (*αhν*) = *A* (*hν* – *E*_*g*_)^*n*^ in this equation, *A* is the constant, *hν* is the photon’s energy, and *n* is the absorption index (Tholkappiyan and Vishista 2014). The optical band gap values of TiO_2_ and (Er^3+^/TiO_2_) NCs were determined using the optical absorption coefficient (*α*). E_g_ values for pristine TiO_2_ and (Er^3+^/TiO_2_) NCs, respectively, reveal that erbium doping affects the band gap energy value of titanium nanoparticles in this study. Because of the Er^3+^ doping, the band gap is reduced, and less energy is required to move an atom from one side of the valence band to the other (Venkatachalam et al. 2019). However, following Er^3+^ doping, the band gap widens. Band gap changes as a result of dopant concentration (Lin et al. 2006). The dopant also prevents electron–hole recombination. However, the effect of a dopant on the band gap is dependent on some parameters, including phase structure, particle size, and the type of the doped component (Ilkhechi and Kaleji 2016).

### Performance evaluation of photocatalytic process

#### Photocatalytic activity of degradation of methylene blue

**Fig. 12:**
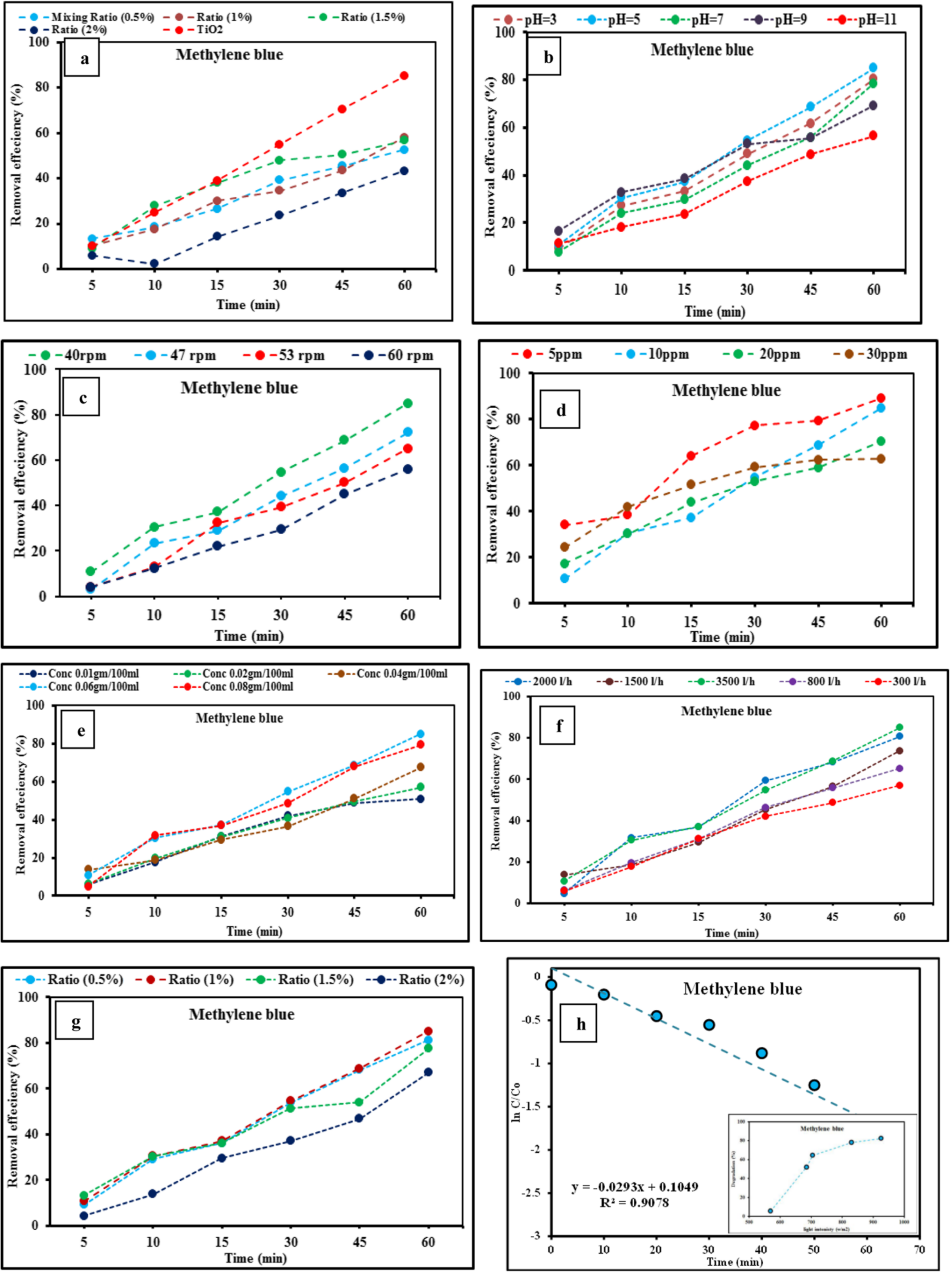
Factors affecting on photocatalytic degradation of methylene blue using pure TiO_2_ and Er^3+^/TiO_2_ NCs. **a** Effect of pure TiO_2_ and the doped ones under UV light. **b** Effect of pH of methylene blue under visible light. **c** Effect of flow rate of motor speed (rpm) of methylene blue under visible light. **d** The effect of dye concentrations under visible light. **e** Effect of different dosing concentrations of catalyst under visible light. **f** Effect of oxygen content by aeration pump under visible light. **g** Effect of different ratios of catalyst under visible light. **h** Effect of light intensity under visible light. All the experiments were done at room temperature (25 °C) and with interval time ranged from 0 to 60 min

When the organic component achieves equilibrium, the UV light is activated depending on the type, concentration, and rate of flow of the organic constituent. Once the system has reached a steady state, the outlet sample is manually collected every 15 min. For 1 h under UV lights, the photoreactor is rinsed with distilled water. The photocatalytic elimination of methylene blue is carried out at a built-up scale utilizing a pilot machine, with a flow rate of 3 L/h of sample, using methylene blue as a wastewater sample. The photocatalytic activity of the produced TiO_2_ particles was determined by monitoring the rate of MB breakdown in an aqueous solution when exposed to UV light. Pristine TiO_2_ made by the sol–gel method without any dopant was used as a reference system. Under UV light, it was found that pristine TiO_2_ only showed higher photocatalytic activity than the doped ones, with degradation efficiency reaching 85% after 1 h (Fig. 12a). The (Er^3+^/TiO_2_) NCs photocatalysts were investigated for the degradation activity under solar exposure. The results were produced by varying parameters such as the pH of the solution (3–11), the flow rate of the motor speed (3–9 L/h at 40–60 rpm), the concentrations of MB (5–30 mg/L), the concentrations, of TiO_2_ doped with erbium (0.01, 0.02, 0.04, and 0.06 wt%), and the effect of different molar ratios of Er^3+^ doped with TiO_2_ (0.5%, 1%, and 2%). Figure 12f shows the impact of an aeration pump on photocatalytic efficiency. The experiments were conducted with interval times ranging from 0 to 60 min because photocatalytic degradability is significant. As the erbium loading was increased, the photodegradation efficiency under visible light increased until it reached 1 mol% and then decreased.

The pH of an aqueous solution is a significant element in the evaluation of photocatalytic activity. By varying the initial pH value while keeping all other variables fixed, we were able to control the pH effect. Photocatalytic activity is reliant on the initial pH of the catalyst and adsorbent, which influences the separation of active functional sites. As demonstrated in Fig. 12b, the dye removal rate increases with increasing pH, reaching a maximum degradation efficiency of 77% at pH 5, with all other parameters held constant, as illustrated in Fig. 12b. The molar ratios of Er^3+^ doped with TiO_2_ are 1% (Er^3+^/TiO_2_) NCs and the amount of catalyst is 0.06 wt% of (Er^3+^/TiO_2_) NCs. The exterior charge of TiO_2_ nanoparticles and the catalytic reaction potentials are shifted due to the pH difference between the contaminants. The Er^3+^ doped TiO_2_ photocatalyst surfaces can be deprotonated or protonated in alkaline or acidic conditions. A stronger oxidizing activity is reported for TiO_2_ at lower pH; however, adding the H^+^ at very low pH can slow the reaction (Gaya and Abdullah 2008).

Methylene blue removal efficiency can be affected by residence time, so four different flow rates (of 3, 4, 6, and 9 L/h, respectively, at 40, 47, 53, and 60 rpm) were tested throughout a range of time intervals from 0 to 60 min. The flow rate and speed of the feed pump are largely responsible for the photocatalytic efficiency, which is controlled by a panel of electricity with a variable speed drive. As demonstrated in Fig. 12c, the photocatalytic effectiveness increases at a low motor speed of 40 rpm (3 L/h) with degrading efficiency of 80%. Photocatalytic degradation is reduced by raising the flow rate because the photocatalytic reaction is accelerated by increasing the flow rate of the feed solution. As a result, the degrading efficiency decreased with an increase in flow rate. The photocatalytic efficiency is inversely related to the feed flow rate. At reduced flow rates, a number of photoactive sites remain available for the reaction to occur. Degradation can be clearly shown to be influenced by flow rate. When the feed water flow is low, the oxygen species are able to interact with the catalyst surface much more easily. Solar light intensity can also be improved using a flow of feed water. In contrast to this, increased flow rates result in an increase in feed water flow, which makes it more difficult for photons to reach the catalyst surface. A higher mass diffusion is also indicated by raising the input flow rate. Contaminant degradation improved in particular with increased mass diffusion and electron–hole recombination (Petit et al. 2007; Assadi et al. 2014).

It was determined that the degradation efficiency (%) of methylene blue (MB) dye could be affected by the initial concentration, which ranged from 5 to 30 mg/L utilizing (Er^3+^/TiO_2_) NCs at a flow rate of 40 rpm (3 L/h) and pH of 5. Figure 12d shows that as the concentration of MB pass away from 5 to 30 mg/L, the rate of degradation slowed down until it reached 70%. The degrading efficiency rises in direct proportion to the MB concentration and exposure time. This may be because the photogenerated holes donate electrons to the adsorbed ^**–**^OH and H_2_O molecules, leading to the formation of the ^•^OH radicals. The generation of ^•^OH radicals, which react fast with aromatic ring compounds, could influence the rate at which the reaction’s rate determination step occurs (Neppolian et al. 2002).

Figure 12e shows the photo-degradation results using Er^3+^-doped TiO_2_. The operating parameters included a dye concentration of 10 mg/L, reaction duration of 0 to 60 min, and a pH of 5. As catalyst quantity improved, it was thought that an increase in the number of catalyst particles would increase dye molecule trapping and photon absorption numbers. Figure 12e shows that the photo-degradation reaction can be accelerated by increasing the amount of catalyst to 0.08 wt%, with a degradation efficiency of 81%. Organic pollutant degradation is caused by an increase in the number of ^•^OH radicals which can be ascribed to the increased amount of catalyst on the photocatalyst surface (Wang et al. 2009).

Figure 12f illustrates the estimation of various periods from 0 to 60 min using an aeration pump through a feed solution with varying flow rates ranging from 300 to 3500 L/h. The aeration increased from 300 to 3500 L/h resulting in an increase in the number of active spots on the reaction medium, which results in an increase in the degradation efficiency from 75 to 84% (Fig. 12f). It was decided that increasing the amount of oxygen bubbled into the feed solution would be preferable for the degradation of MB. Reactive oxygen species such as O_2_^•^, H_2_O_2_^•^, and ^•^OH radicals were created as a result of the aeration pump, which increased the efficiency of the process. The photocatalytic activities of these extremists are responsible for the degradation of organic pollutants, the destruction of harmful organic contaminants, and the oxidation of structures (Assadi et al. 2014). To avoid electron–hole pair recombination, an aeration pump was incorporated into the current photo reactor (Sun et al. 2009). Degradation by photocatalysis was completed by oxygen pumping into the feed solution. Er^3+^ ions may not be giving off much light because they are interacting with the TiO_2_ host. Er^3+^ ions could give some energy to the TiO_2_ conduction band (Reszczyńska et al. 2015).

An experiment performed on variations of five light intensities (569 W/cm^2^, 685 W/m^2^, 705W/m^2^, 830 W/m^2^, 925 W/m^2^) observed that the degradation of MB enhances by increasing the intensity of light. Improvement of the degradation rate by increasing the light intensity was also monitored. Figure 12h indicates an increase in dye degradation due to an increase in intensity (Kundu et al. 2005). The photo-decolorization of dyes on (Er^3+^/TiO_2_) NCs was increased by increasing the light intensity. With increasing light intensity, the decolorization of a dye is affected by both light intensity and exposure time (Gaya and Abdullah 2008). Photocatalysts absorb light energy equal to or greater than the energy of the band gap, resulting in the transfer of electrons from VB to CB and the formation of holes in VB (Sohrabnezhad et al. 2009). By increasing photon intensity, the rate of photocatalytic degradation increases as well (Gao et al. 2011). Increasing the intensity of incident light increases the probability of photocatalyst excitation (Saleh et al. 2011).

Under visible light, the photocatalytic activity of (Er^3+^/TiO_2_) NCs was shown to be significantly higher than the photocatalytic activity of TiO_2_. Increasing the Er^3+^ doping dosage initially boosted the photocatalytic activity of TiO_2_-based catalysts; however, when Er^3+^ doping content was greater than 1.0% when illuminated by visible light, this activity reduced. Under visible light, 1.0% (Er^3+^/TiO_2_) NCs had the best photocatalytic performance (Fig. 12g). When the methylene blue absorbs visible light, electrons are generated and can be activated. After that, the excited electron can be turned into TiO_2_, and then it can be converted into O_2_, which will result in O_2_^•^ and a hydroxyl radical, as shown in Eqs. (1)–(6). Therefore, pristine TiO_2_ should have photocatalytic activity owing to dye sensitization (Liang et al. 2006).

1 $$\mathrm{Dye}+\mathrm{hv}\to {\mathrm{dye}}^{*}$$

2 $${\mathrm{TiO}}_{2} +\mathrm{ dye}*\to {\mathrm{ TiO}}_{2} ({\mathrm{e}}^{-}) + {\mathrm{dye}}^{\cdot +}$$

3 $${\mathrm{O}}_{2}+{\mathrm{TiO}}_{2}\left({\mathrm{e}}^{-}\right)\to {{\mathrm{O}}_{2}}^{\cdot -}+{\mathrm{TiO}}_{2}$$

4 $${{\mathrm{O}}_{2}}^{\cdot -}+{\mathrm{H}}_{2}\mathrm{O}\to {{\mathrm{HO}}_{2}}^{\cdot }+{\mathrm{OH}}^{-}$$

5 $${{\mathrm{HO}}_{2}}^{\cdot }+{\mathrm{H}}_{2}\mathrm{O}+{\mathrm{TiO}}_{2}\left({\mathrm{e}}^{-}\right)\to {\mathrm{H}}_{2}{\mathrm{O}}_{2}+{\mathrm{OH}}^{-}+{\mathrm{TiO}}_{2}$$

6 $${\mathrm{H}}_{2}{\mathrm{O}}_{2}+{\mathrm{TiO}}_{2}\left(\mathrm{e}-\right)\to { }^{\cdot }\mathrm{OH}+{\mathrm{OH}}^{-}+{\mathrm{TiO}}_{2}$$

#### Photocatalytic reaction mechanism

**Fig. 13:**
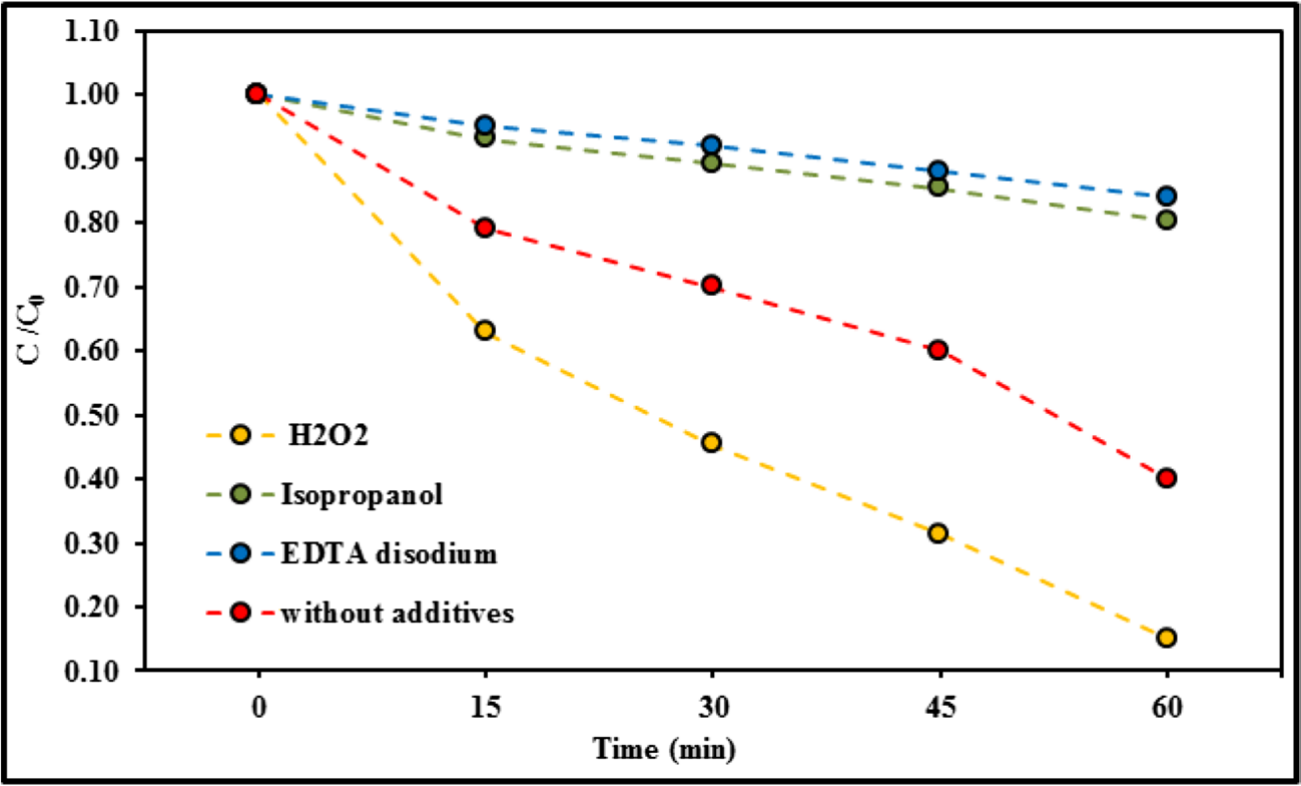
Effects of different scavengers on the photo-degradation of MB using (Er^3+^/TiO_2_) NCs catalyst. Reaction conditions: 0.001 g Methylene blue in 100 ml deionized water, 0.1 ml H_2_O_2_, 0.06 g catalyst at room temperature

The results of different scavengers on the photo-degradation of (Er^3+^/TiO_2_) NCs under visible light irradiation were examined to determine the reactive oxygen species that control the catalytic action (Fig. 13). The ^•^OH was scavenged via using isopropanol (IPA) (10 mM), where h^+^ was scavenged with EDTA disodium (5 μM).To determine the effect of the absence of an electron scavenger on MB degradation operation, a single set was run without H_2_O_2_, as shown in Fig. 13. Following the addition of the above additives, the catalytic degradation efficiency of (Er^3+^/TiO_2_) NCs against MB was altered, denoting the sequence: EDTA disodium < IPA < without additives < H_2_O_2_. As a result, after removing H_2_O_2_, the degradation of MB using (Er^3+^/TiO_2_) NCs was significantly reduced (Fig. 13). This elucidates the role of electrons and oxygen peroxide radicals in the oxidation of MB underneath the visible-light illumination since the presence of electrons allowed the reduction of H_2_O_2_ to create ^•^O_2_^−^, as shown below:

7 $${\mathrm{H}}_{2}{\mathrm{O}}_{2}+{\mathrm{e}}^{-}{2\mathrm{H}}^{+}+{ }^{\cdot }{\mathrm{O}}_{2}$$

Other scavengers, such as isopropanol and EDTA disodium, had much lower impact on MB degradation, implying that the most active radicals responsible for MB photo-degradation were electrons and ^•^O_2_^−^, with recording that HO^•^ and h^+^ had much lower effects. The minimal role of HO^•^ in the degradation course may possibly ascribed to the scavenging reaction of parts of the constructed HO^•^ by H_2_O_2_ as follows:

8 $${\mathrm{H}}_{2}{\mathrm{O}}_{2}+{\mathrm{h}}^{+}\to {\mathrm{H}}^{+}+{{\mathrm{HO}}^{\cdot }}_{2}$$

9 $${{\mathrm{HO}}^{\cdot }}_{2}+{\mathrm{HO}}^{\cdot }\to {\mathrm{H}}_{2}\mathrm{O}+{\mathrm{O}}_{2}$$

10 $${\mathrm{H}}_{2}{\mathrm{O}}_{2}+{\mathrm{e}}^{-}\to {2\mathrm{H}}^{+}+{ }^{\cdot }{\mathrm{O}}_{2}$$

## Conclusion

In conclusion, (Er^3+^/TiO_2_) NCs were manufactured via sol–gel synthesis to maximize photodegradation under visible light. The FT-IR of Er–O bond confirmed Er^3+^ doping with TiO_2_. Erbium ion doping improved anatase TiO_2_ thermal stability, reduced crystallite formation, and increased surface area. Surface and structural nanoparticle properties were evaluated using SEM and EDX. The Er, Ti, and O are agglomerated and irregularly dispersed. The XPS was used to study Ti, Er, and O surface electrical states. Er^3+^ ion concentration affected hydrodynamic nanoparticle size. Negative zeta potential indicates nanoparticle stability and high dispersion. The catalysts’ higher specific surface area enables improved substrate adsorption. The Er^3+^ dopant decreases the band gap, allowing electrons to travel from the valence to the conduction band more easily. To evaluate the efficiency of produced nanoparticles and the continuous photoreactor, many aspects were considered. The pH of feed water, motor flow rate, catalyst ratio, pollutant types, and concentrations were considered. Pristine TiO_2_ only showed increased photocatalytic activity under UV light. The pH5 was enhanced degradation efficiency. Feed water flow is modest at low flow rates. Oxygen species contact the catalytic surface more often. Having enough feed water also increases solar light intensity. Increasing MB from 5 to 30 mg/L lowered decomposition efficiency. The photodegradation rate increases with catalyst quantity and peaks at 2%. More oxygen from the aeration pump increased system performance. Increasing the air pump’s speed increases the amount of oxygen bubbling into the feed solution, improving pollutant removal efficiency. The Er^3+^/TiO_2_ NCs had greater photocatalytic activity than pristine TiO_2_ for MB degradation. This is because rare-earth ions and anatase TiO_2_ have a synergistic effect that improves light absorption and prevents photogeneration electron–hole pair recombination.

## Data Availability

The datasets used and/or analyzed during the current study are available from the corresponding author on reasonable request.
